# Quality and safety issues highlighted by patients in the handling of laboratory test results by general practices–a qualitative study

**DOI:** 10.1186/1472-6963-14-206

**Published:** 2014-05-06

**Authors:** David Edward Cunningham, Duncan McNab, Paul Bowie

**Affiliations:** 1 Postgraduate GP Education, NES Education for Scotland, 2 Central Quay, 89 Hydepark Street, Glasgow G3 8BW, UK

## Abstract

**Background:**

In general practice internationally, many care teams handle large numbers of laboratory test results relating to patients in their care. Related research about safety issues is limited with most of the focus on this workload from secondary care and in North American settings. Little has been published in relation to primary health care in the UK and wider Europe. This study aimed to explore experiences and perceptions of patients with regards to the handling of test results by general practices.

**Methods:**

A qualitative research approach was used with patients. The setting was west of Scotland general practices from one National Health Service territorial board area. Patients were purposively sampled from practice held lists of patients who received a number of laboratory tests because of chronic medical problems or surveillance of high risk medicines. Focus groups were held and were audio-recorded. Tapes were transcribed and subjected to qualitative analysis. Transcripts were coded and codes merged into themes by two of the researchers.

**Results:**

19 participants from four medical practices took part in four focus groups. The main themes identified were: 1. Patients lacked awareness of the results handling process in their practice. 2. Patients usually did not contact their practice for test results, unless they considered themselves to be ill. 3. Patients were concerned about the appropriateness of administrators being involved in results handling. 4. Patients were concerned about breaches of confidentiality when administrators were involved in results handling. 5. Patients valued the use of dedicated results handling staff. 6. Patients welcomed the use of technology to alert them to results being available, and valued the ability to choose how this happened.

**Conclusions:**

The study confirms the quality and safety of care problems associated with results handling systems and adds to our knowledge of the issues that impact in these areas. Practices need to be aware that patients may not contact them about results, and they need to publicise their results handling processes to patients and take steps to reassure patients about confidentiality with regards to administrators.

## Background

In the United Kingdom (UK) and internationally, general practitioners (GPs) arrange large numbers of laboratory tests, radiological and other investigations for patients in their care. The UK Quality and Outcomes Framework of the GP Contract expanded primary healthcare’s involvement in the management of many long-term conditions increasing the number of investigations performed by general practices [1]. This is likely to continue given the Scottish Government’s vision that primary healthcare teams will undertake more complex work in partnership with other community agencies [2,3].

In order to cope with the increasing complexity and volume of primary healthcare work, administrators have developed additional skills and responsibilities such as co-ordination of repeat prescribing systems, undertaking phlebotomy and healthcare assistant duties [4-6]. In addition, they are often involved in the handling and communication of test results to patients which carries a significant, often safety-critical risk.

Primary healthcare based research and improvement studies concerned with the management of laboratory tests results have demonstrated the potential for patients to be avoidably harmed as a consequence of inadequate systems, including the communication processes for informing patients of test result outcomes and necessary follow-up actions [7,8]. A significant proportion of all medico-legal claims in primary healthcare are related to delayed, missed or inaccurate diagnoses with unsafe and ineffective laboratory test ordering and results management systems frequently cited as contributory factors in these failures [9].

However, much of the patient safety work is limited to the study of USA and European secondary healthcare systems [7,8,10-14]. Overall, there is a paucity of related research in the United Kingdom (UK) and wider Europe despite the widespread recognition of the safety-critical nature of this issue in the evidence base and by medical indemnity organisations. Much of the aforementioned research has focused on the critical review of organisational systems, and of the perceptions and experiences of clinicians and healthcare managers on what can go wrong [8,15-18]. The views and experiences of patients who routinely interact with frontline primary healthcare staff when attending for investigations and re-attending for results are limited to a small number of studies, but none has been undertaken in the European context. The critical importance of the voice of patients in contributing to patient safety research and improvement is lacking, particularly on this issue where they are able to directly observe and experience potential and actual human-system errors and their consequences on a daily basis [19].

There is a lack of evidence on how patients’ experiences of interacting with the practices’ results handling systems impacts upon safety. Patients’ understanding of the responsibilities of healthcare professionals and staff and their experience as partners in the communication of results is required to help inform safe and effective systems [20]. The aim of this study was to identify the perceptions and experiences of patients with respect to the handling and communication of test results in primary healthcare.

## Methods

A qualitative research approach was adopted to explore the perceptions and experiences of patients. Focus groups were selected as a data collection method as they encourage interactions amongst participants, and allow for the exploration of perspectives and understanding about a topic [21]. The study consisted of a recruitment stage, data collection, and data analysis undertaken by DC and DM, two experienced GPs who held educational roles within NHS Education for Scotland (NES), a special health board with responsibility for the education and training of the NHS workforce in Scotland.

### Recruitment stage

A purposive sampling strategy was adopted in order to achieve maximum diversity of perceptions and experiences. DC identified general practices from one NHS board. It was considered that practice size may influence how results were handled by general practices. Practices were stratified into three groups: small (up to 5,000 patients) medium (between 5,001 to 10,000 patients) and large (greater than 10,000 patients). Practice managers were sent a written invitation to assist with the study and were asked to recruit patients from their practice who had long term conditions or who underwent monitoring of high risk medicines. It was considered that these patients would have considerable experience of interacting with the practice’s test results handling processes. Practice managers were asked to recruit one focus group each, and to stop when eight patients had been recruited.

### Data collection

Participants were given an information sheet by DC outlining details of the study aims. Each participant signed a consent form given by DC allowing their discussion to be audio-recorded and transcribed. An assurance was given that discussions would be confidential and the transcriptions would be anonymised. Focus groups were held in the patients’ own practices, rooms used were private and confidential. The groups were moderated by DC who is an experienced moderator. An interval of two weeks between each focus group allowed for an iterative process to be adopted, and for emergent themes from one focus group to be presented and considered by later focus groups. The following question topic guide was used: (Table 1).

**Table 1:** Question topic guide

1	How do you access your test results from the practice?
2	How easy is it to get your results from receptionists?
3	Has it ever gone wrong in the past?
4	What happens when you make contact with the practice for your test results?
5	How could it be improved?

### Data analysis

Transcriptions were checked against original audio-recordings and corrections made. Transcripts were read and codes were constructed using thematic analysis [22]. Codes were then merged with others to form themes. Coding was undertaken independently by DC and DM who then discussed and negotiated the construction of themes.

### Ethical approval

This study was pre-screened by the West of Scotland Research Ethics Committee and was judged to be service evaluation.

## Results

### Focus group participants

Four focus groups were held comprising a total of 19 participants with 13 being female. Group size ranged from four to seven participants. Participants ranged in age from 45 to 80 years. Meetings lasted between 60 and 90 minutes. One focus group was drawn from a small practice, two from medium sized practices and one from a large practice. Two practices recruited participants with long term conditions who were involved in the practice’s Patient Participation Group. These participants held regular meetings with the practice manager and were experienced in giving constructive criticism and feedback from a patient’s perspective about ongoing quality of care issues in the practice. Data saturation was achieved by the third focus group, a fourth group was undertaken to confirm this (Table 2).

**Table 2:** Main themes from focus groups

1	Lack of awareness of results handling processes
2	The communication of results
3	Appropriateness of administrators’ involvement in results handling
4	Concerns about confidentiality
5	Dedicated results handling staff
6	Administrators’ use of technology to inform patients of test results

### Lack of awareness of results handling processes

A dominant theme from all four focus groups was participants’ lack of awareness of the processes involved in results handling. Whereas most participants were able to envisage their test sample being transported to the laboratory for analysis, few knew how their test results were returned to their practice and how results were handled by different staff groups.

“*But we don’t know how the system works. When the results come back from the hospital to the surgery, who processes those results? Is it the receptionist gets a great big long email from the hospital, and she puts it into the machine? Or do they go to the doctor and he puts it in or…?*” (Group 3, participant 3)

Some participants were unsure of who was involved in the handling of test results but felt this should be solely in the domain of clinical staff such as GPs and practice nurses. A number of participants perceived that administrators had little involvement in test results handling processes, and that they were unlikely to be able to access their test results or to see their medical files. As a consequence, some participants were surprised when they received a letter or telephone call from an administrator alerting them to test results.

“*If there’s something wrong with you I, we’re going back to the doctor ringing and telling you, I would have thought something like that would come straight from the doctor, not via the receptionist.*” (Group 4, participant 2)

In contrast, others considered that primary healthcare clinicians would need assistance with large numbers of test results returning to each practice, and that the involvement of administrators made results handling more efficient and effective. An opinion was given by one participant who had experience of the workload from his role within the NHS:

“Working in the hospital I know the volume of tests that goes up to the ******* [local hospital] *labs and they would need to employ somebody full-time and I’m, not sort of, I’m just saying how it is. The volume of tests that goes to the labs is unbelievable. We have vans go round the surgeries and pick them up three times a day from all the surgeries.*” (Group 3, participant 4)

Some participants considered they lacked information or clarity about how they were expected to access their test results from the practice and they perceived this resulted in a degree of confusion about their role and responsibility.

### The communication of results

Most participants reported that they did not contact the surgery to access their results and felt that clinical staff would contact them directly with significantly abnormal test results. This was regarded as a default position by participants and most reported that they relied on clinicians to contact them about abnormalities identified in tests arranged by the practice. Participants recalled how GPs contacted them at home by telephone, or telephoned their mobile phone to alert them to significant test results.

“*But normally my GP just phones up and says: ‘This is what’s happened. Could you come and see me?’ Or whatever.*” (Group 1, participant 4)

“*If there’s something they find adverse in the test that they give you, then they call you*.” (Group 3, participant 1)

A few participants considered that patients had a responsibility to find out test results and to ensure that these were being actioned by the practice. The perceptions of being ill, or of feeling unwell, emphasised the importance of patients making contact with the practice. Indeed, the more unwell a patient felt, then the greater responsibility that patient was perceived to have in making contact with the practice to determine the significance of recent tests:

“*If I’ve had blood taken and it’s a routine check and I don’t hear anything from the surgery I’m quite happy just to let it go until the next time I’m in. But if I’m unwell and I come and have blood taken or something like that then I, at the moment, it’s my responsibility to phone the surgery.*” (Group 3, participant 4)

Patients who had participated in some years of routine monitoring of chronic disease or of high risk medicines expected the practice to make contact about any abnormalities found. There was a perception that “no news is good news” and that participants considered that if no contact was made by the practice, then results were assumed to be within normal limits.

“*There must be a lot of people who don’t phone in for results, personally I don’t. If I get a blood test taken I just forget about it. I just wait for them, the receptionists to phone me, and hope they don’t.*” (Group 3, participant 1)

Most participants conceded that they did not follow up their own test results, especially if they considered they were in good health. They were confident that the practice would make efforts to contact them about significant results that required further action:

“*And I’m afraid I’m a bit, ehm, when I’m told to phone in for the results and I kind of forget that I was told to phone. If there’s anything if there’s any problems I get a phone call from the doctor.*” (Group 1, participant 4)

*“My maxim is if I don’t hear from them* [medical practice] *it’s not urgent. So I don’t usually bother phoning in now.*” (Group 4, participant 4)

For a number of participants, their lack of personal follow up reflected difficulties getting through on busy telephone lines, and restrictions on hours of telephone access for test results.

### Appropriateness of administrators’ involvement in results handling

A number of participants raised concerns about the competence of administrators in their involvement in results handling. There were recurring concerns that administrators had little or no clinical training, and that involving them in results handling stretched them beyond their capabilities, and as a consequence created patient safety issues.

“*There was just one incident, the ehm, the receptionist on the phone said: ‘The results were borderline and could I come back in a month?’ Anyway, but when I went to the nurse fortunately I had an appointment made for about ten day’s time after that. I went back and I said to the nurse: ‘Oh! I just kept this anyway. I had to, I was told to come back in a month.’ And she was really quite disturbed by that and said I should have been back in a week.*” (Group 2, participant 5)

Easier access to administrators for test results was counter-balanced by their lack of clinical knowledge; participants judged that enquiries regarding the interpretation of results put administrators under strain. The qualifications and training experience of administrators were not known to most participants and all focus groups raised this issue as a concern. A small number of participants recalled how they or their family had been given inappropriate or wrong clinical advice by administrators.

“*My concern would be is that I don’t know, I’m assuming receptionists are not medically trained, so therefore what you’re talking about is communication and the receptionist should not put any interpretation on the results like say the lady* [other focus group participant] *whose bloods are low. In my opinion, receptionists shouldn’t be doing that, that should come from a nurse or doctor or somebody with some* [clinical experience].” (Group 2, participant 4)

Some participants considered that the involvement of administrators in results handling was unfair for this staff group in that they were being placed in awkward situations with patients and that they were involved in the clinical management of patients inappropriately. A number of participants considered that administrators should be given specific messages or instructions from clinical staff to communicate to patients and that they had no role in the interpretation of test results:

“*Yeah, I think I assume that the receptionist will have been informed by a doctor that the results that they were going to tell you, what to tell you. Not to make up their own dialogue from what they assume.*” (Group 1, participant 4)

“*Yes, I think that would bother me if it was, ehm, they* [administrators] *were interpreting the results but I would imagine it would be as it is now, that it goes to the doctor first.*” (Group 1, participant 2)

*“It’s okay maybe for a minute or so and then the receptionist, I can just visualize the receptionist would get so muddled up in my opinion. You know, about what progress has to be made, you know, medical progress has to be made here, and I just feel from the patient’s point of view I don’t think that’s acceptable. It would be dumbing the whole process down.*” (Group 1, participant 4)

The role of administrators in communicating abnormal test results was considered appropriate if it involved contacting patients to make follow-up appointments: either by telephone or in person with the clinician involved. The breaking of bad news was considered to be a task for clinicians only, and that administrators could only be involved in sharing results that showed no concerns or minimal abnormalities.

“I agree with ****** [focus group participant], I don’t think that’s the role of a receptionist. No disrespect to them but I don’t think that’s really the role of a receptionist. And for ******’s explanation that you know they’re not, well, they’re not doctors you know, and some confusion and mistakes could be made along the lines, and I think you’ve got to watch that.” (Group 1, participant 2)

“*I think the real danger here is we’re asking people* [administrators] *to interpret or potentially interpret, I think there’s a huge danger in that, for me, just text me them and I’ll call in for an appointment*” (Group 2, participant 7)

### Concerns about confidentiality

A dominant theme from all four focus groups related to confidentiality and this involved administrators in three ways. A few participants felt their test results were confidential between them and the relevant clinician involved, and that test results processes should not involve administrators as it would be an invasion to patient privacy. These participants did not envisage that administrators should have access to private or confidential information about themselves. They considered it appropriate that administrators could make contact with them, but did not want them to be aware of their results, nor the context of that result. A number of participants considered that the administrator’s role was to assist clinicians with tasks such as arranging appointments or telephone calls to the clinician, or acting as a communication channel.

“*I was talking to somebody last night about this, and I said I didn’t agree with the receptionist doing it, because as far as I’m concerned it’s personal.*” (Group 3, participant 2)

The second confidentiality issue related to participants’ fears that their test result would be made known to others by administrators. This was either in error to a mis-identified patient or relative, or when administrators contacted patients on their household’s telephone number. Participants were also concerned about messages regarding test results being left on a home answering machine as there was a risk that others in their household could learn of the result.

“*I don’t like that either. If the message is left on the answering machine, because anyone could get your message then. It could be your daughter, your eh, your daughter’s boyfriend.*” (Group 3, participant 4)

The third confidentiality issue related to test result information being overheard by other patients at the reception desk. Some participants recalled hearing administrators informing patients of test results, and they had overheard such conversations from the waiting room. They were unhappy about this and considered it a significant breach of confidentiality. One practice had forbidden their staff from informing patients of test results at reception, and although this safeguarded confidentiality, it reduced access to test results. Participants were aware that incoming telephone calls from patients could be answered by receptionists near to waiting patients, and that this could result in breaches in confidentiality.

“*I think that it’s pretty open actually out there* [waiting room/reception area] *as well and when you’re sitting, somebody phones up maybe a prescription or another repeat prescription and you hear:* ‘*What’s your name, your address what was that?*’ *And they say* ‘******* Road.’ *You know you can hear all that and I think it’s pretty open.*” (Group 3, participant 4)

“*I don’t think that’s right. If you want to speak privately there should be a facility where you can go and somebody will come and speak to you privately.*” (Group 3, participant 2)

### Dedicated results handling staff

One practice had centralised results handling within the practice and had a dedicated telephone line and staff. These staff members dealt with all patients who wished to obtain their results. A number of benefits of this working method were praised by participants in the first focus group. Participants envisaged that the ‘results telephone line’ was answered in a practice area away from public areas and there were fewer concerns about inadvertent breaches of confidentiality.

“*But it’s handy because it’s a results line so that’s not at the front desk for everybody listening.*” (Group 1, participant 2)

The results telephone line was a different telephone number from the practice’s main switchboard and participants considered they were not competing with patients calling the practice for appointments. There were perceptions that administrators who answered the results line were more experienced in the communication of test results and would be able, in a limited way, to help patients gain more information about their results.

“*I like to get the value* [long term marker of glycaemic control] *you know. That’s never been denied me, it’s just I’ve had the receptionist, maybe doesn’t have it to hand at the call. I say if you don’t mind you know 'cause I keep a very careful record of these things. I mean it’s nice to know it’s satisfactory, but I like to know if it’s up or down. So she gets these figures and passes them to me on my phone and I’ve never had difficulty with it at all.*” (Group 1, participant 3)

Participants perceived that trained results handling staff would be better equipped to give further details about test results and that their telephone call would not be as rushed as administrators answering the main telephone number. Participants considered that administrators were often in a hurry to terminate their call, because of the pressure to answer incoming telephone calls, and that this resulted in quick and superficial results handling.

“*There was a concern in my bloods and my sister said: ‘Remember and phone in.’ And I did. So she* [sister] *said:* ‘*Sometimes it’s how quick they give you the results. They’ll say: ‘Everything’s fine.*’ *Now they have no way of checking off what they have to check. Everything’s fine. So then I said* [to receptionist]*:* ‘*So, could you tell me what this reading is?*’ *She* [receptionist] *went*: *‘I don’t know your, what that is.’*” (Group 3, participant 6)

Others perceived that dedicated results handling staff might have closer relationships with relevant clinicians and this would improve the interpretation and communication of test results. Participants felt that this small team could develop their own communication skills and be more likely to check that patients had understood their test results.

“*As long as they’re trained. As long as they’ve undergone training, I think that’s important.*” (Group 1, participant 1)

“*It would be a good idea* [creation of a results line] *and I think it would be safe enough. Particularly if you’re dealing with two receptionists, let’s say, who are more au fait with the information coming through.*” (Group 2, participant 7)

### Administrators’ use of technology to inform patients of test results

Participants acknowledged that there were a number of communication methods that practices could use to make contact about test results. Mobile telephones and their answering machines, text messages, email and online access to results were discussed. Most participants felt that technology would be useful to inform them that a result was available but did not wish to have the actual result communicated to them in this way. The option of using a mobile phone was broadly welcomed and participants envisaged that administrators would have a significant role to play in this. Participants perceived that mobile phones could improve confidentiality and were significantly better than home answering machines.

Two of the four practices were using text messages to remind patients about booked appointments and this was well received by participants. These groups felt that extending this method into results handling would be effective and welcomed by patients. A few patients felt that the use of mobile phones and the sending of text messages were impersonal and damaging to the relationships between clinicians and patients.

“*I’m no[t] sure that that’s something that I would be terribly comfortable with. What’s wrong with texts is that people don’t talk.*” (Group 4, participant 2)

“*Well I think it should be, I think important for everyone is this relationship you might say, between the doctor and patient. Presumably your usual doctor, you know and I wouldn’t want to see that type of relationship sort of broken or interfered with in some way. And that’s become a danger, I’m all for technology, but I can see there may be dangers ahead, you know, with using that.*” *(Group 1, participant 3)*

Some participants considered that online access to their medical notes would be beneficial, as long as test results were interpreted by clinicians before they could be seen by patients. The ability to choose a method of accessing test results was valued by participants, and they appreciated being able to select a method from a range on offer to them, and that this could be decided at the end of the consultation with the clinician.

“*Is this not saying that perhaps the patient should be asked how they wish to be communicated? Rather than one set way, you have a range of ways the patient can say how they want to communicate.*” (Group 2, participant 6)

“*Yeah, but so perhaps there is some swipe cards that everybody can have, you know, like a bank card and you stick it in the machine somewhere, and you can get the printout of your results or something. There might be something that these designers can come up with that’s going to make your life easier in the future.*” (Group 1, participant 1)

## Discussion

### Summary of main findings

This study identified a number of themes from patients about the quality and safety of care related to how test results are handled and communicated by general practices. Patients had limited knowledge of the results handling processes involved, and of how results would be communicated to them. Patients were concerned about the appropriateness of administrators being involved in the handling processes and expressed concerns about confidentiality issues. Some said that dedicated results staff would improve safety and effectiveness, and that the use of technology such as mobile phones and texting should be offered to patients.

### Strengths and limitations

There were a number of strengths of the study. Participants had considerable experience of having tests performed in general practice and of accessing test results over a number of years. They were able to draw on those experiences to consider safety and quality issues in results handling. Participants were recruited from practices of varying size, from four towns and villages within one Scottish NHS board, and data saturation was achieved. Two focus groups were drawn from Patient Participation Groups and these patients were experienced in giving focused feedback on a number of issues relevant to general practices [23].

The qualitative study design allowed participants to express their perceptions and experiences freely, and its iterative nature allowed for emerging themes to be examined and considered by subsequent focus groups.

The study had a number of weaknesses: participants were recruited from only one Scottish NHS board, and they were all over 45 years old. The views of younger patients and those from other UK areas may be different. Patients who attend their doctor infrequently and do not have regular tests performed may hold different perceptions about the processes involved in test results handling. Patients were recruited by practice managers as no other recruitment method of patients was available; there may be bias associated with this recruitment method. Similarly patients from Patient Participation Groups may be different from the general population. All participants’ first language was English and none had problems with sensory impairment such as blindness or deafness. The study does not reflect fully the geographic and demographic characteristics of all general practice patient populations.

The study adds to service evaluations about the important role of administrators in primary healthcare and to the literature regarding our understanding of the safety and quality of test results systems. Earlier research has drawn attention to breaches of confidentiality in primary healthcare waiting rooms and reception areas, and over the telephone [24-26]. Participants favoured the use of dedicated results staff and telephone lines as a method that may prevent breaches of confidentiality and improve quality of healthcare. The feasibility of general practices in adopting this model is not known. The use of dedicated results staff may have significant workforce implications for smaller practices and might result in de-skilling of other administrators.

Participants described a lack of understanding of the results handling systems used by practices, and may not understand the important role of administrators. Patients should be given a clear description of how they will be informed of their results and who in the primary healthcare team may be involved in this. This could help improve the safety of the results handling system and ensure that results are communicated effectively.

In contrast to research findings from the United States, participants had fewer concerns about the importance of finding out their test results, and there was an assumption that primary healthcare clinicians would always act on abnormal results [27,28]. There may be cultural influences on patient safety issues, and UK primary care clinicians should be aware of their patients’ expectations regarding this (Table 3).

**Table 3:** Issues practices may wish to reflect upon

1	Practices should consider publicising how test results are handled and managed within the practice, and how results may be communicated to patients. Patients may want to make an individual choice in how they receive notification of test results.
2	Clinicians should consider giving explicit information to patients about their own role, and encourage patients to contribute towards patient safety.
3	A results handling telephone line and dedicated staff would be welcomed by patients in this study as it is perceived by them to be safer and more effective.
4	Practices should emphasize to patients that administrators have a duty of confidentiality similar to that of clinicians in the practice.
5	When clinicians delegate the communication of test results to administrators they need to give unambiguous and detailed instructions to prevent harm to patients.

The patient safety research and improvement agendas are limited in primary care, although there is growing interest in both [29,30]. The role of patients’ contribution to the development of safety initiatives is also scarce, which will be incomplete and lacking in credibility if the patients’ perspective is not sought and considered [19]. If we are to take patient safety seriously then we must treat patients as active partners in improvement programmes rather than as passive recipients of healthcare. We should acknowledge that healthcare professionals may be uncomfortable with this prospect and that cultural change may be necessary. Our study has alluded to some of the aforementioned improvement principles as we judged it essential, given the lack of relevant scholarly publications, to capture the experiences of patients who routinely require blood tests and monitoring as one way to contribute their perspectives to the development of evidence-based guidance for laboratory test ordering and systems-based results handling [31].

## Conclusions

The findings from this small study will be useful in informing the next phase of the Scottish Patient Safety Programme in primary care, given the strong likelihood that test result handling will be a selected topic for safety improvement [29]. What is clear from our results is that patients may need more specific information and guidance around how practice systems for managing test results operate, and how the patient could be an active participant in order to improve safety. Future research could focus on patients with less experience of test results handling.

## Competing interests

The authors declare that they have no competing interests.

## Authors’ contributions

PB conceived the study idea, acquired funding and assisted with the study design and critical revision of the manuscript. DC assisted with the study design, led the data collection, analysis and interpretation and co-drafted the manuscript. DM contributed to the data analysis and interpretation and co-drafted the manuscript. All the authors have approved the manuscript.

## Pre-publication history

The pre-publication history for this paper can be accessed here:

http://www.biomedcentral.com/1472-6963/14/206/prepub
